# Seven draft genome sequences of *Streptococcus canis* strains, revealing reduced penicillin-G susceptibility

**DOI:** 10.1128/mra.00219-24

**Published:** 2024-05-14

**Authors:** Yuya Kawara, Mieko Goto, Takahiro Maeda, Haruno Yoshida, Yuzo Tsuyuki, Takashi Takahashi

**Affiliations:** 1 Laboratory of Infectious Diseases, Graduate School of Infection Control Sciences, Ōmura Satoshi Memorial Institute, Kitasato University, Tokyo, Japan; 2 Division of Clinical Laboratory, Sanritsu Zelkova Veterinary Laboratory, Tokyo, Japan; Department of Biological Sciences, Wellesley College, Wellesley, Massachusetts, USA

**Keywords:** *Streptococcus canis*, penicillin-G, reduced susceptibility, penicillin-binding proteins

## Abstract

We report seven draft genome sequences of *Streptococcus canis* strains revealing reduced penicillin-G susceptibility. The genomes measured 2.054–2.385 Mbp, with G+C contents of 38.8%–39.6%. Amino acid substitutions in penicillin-binding proteins were characterized as compared with those of NCTC 12191(T) genome sequence (GenBank accession number NZ_LR134293.1).

## ANNOUNCEMENT

Penicillin-G and β-lactamase inhibitors are the first lines in treating canine deep pyoderma. Three *Streptococcus canis* strains with reduced penicillin-G susceptibility (PRScanis) or penicillin-G resistance are isolated from two different canine skin abscesses or a different oral cavity through sampling by sterile swabs/needle aspiration/biopsy (1). We report seven genome sequences of PRScanis strains. Potential use of the provided genomes characterized amino acid (AA) substitutions in penicillin-binding proteins (PBPs) as compared with those of NCTC 12191(T) genome sequence (GenBank accession number NZ_LR134293.1).

Sanritsu Zelkova Veterinary Laboratory approved the study design (approval no. SZ20230921). We selected seven strains with elevated penicillin-G MICs (μg/mL) by the broth microdilution method within previously reported collections (2–4) and confirmed them to be PRScanis strains with increased MICs (0.06–0.125) by the agar plate dilution method (susceptible MICs < 0.12) based on Clinical and Laboratory Standards Institute document M100-S16 (5) (Table 1).

**TABLE 1:** Seven draft genome sequences of *Streptococcus canis* strains, revealing reduced penicillin-G susceptibility

Strain (host/isolation source/isolation year/geographic location)	Nucleotide accession no. (SRA accession no.)	Genome size (bp)	Number of contigs	Average coverage	*N*_50_ value (bp)	Numbers of genes/CDSs/tRNAs/ rRNAs/CRISPR	G+C content (%)	Coding ratio (%)	Sequence type (clonal complex)^*b*^	M-like protein allele type^*c*^	Acquired antimicrobial resistance genes^*d*^	Penicillin-G MIC (μg/mL)^*e*^
NCTC 12191(T) (bovine/milk/unknown/unknown)	NZ_LR134293.1 (ERR1274812)	2,084,744	1		2,084,744	2,056/1,967/67/18/4	40	84.5	ST9	Allele 1	None	<0.03
SA15 (dog/ear discharge/ 2015/Tokyo)	BAAAAO000000000.1 (DRR527837)	2,105,561	43	465×	130,584	2,006/1,968/33/2/3	39.4	85.1	ST2 (CC46)	Allele 2	None	0.06
KU43 (dog/pus/2021/Gifu)	BTXP00000000.1 (DRR518369)	2,064,483	47	1,160×	101,510	1,979/1,934/39/3/3	39.6	84.8	ST46 (CC46)	Allele 2	*ant*(6)-*Ia*, *erm*(B), *tet*(O)	0.125
KU44 (dog/cornea/2021/ Tokyo)	BAAAAM000000000.1 (DRR527834)	2,088,583	68	531×	93,919	1,974/1,939/29/3/3	39.6	83.8	ST46 (CC46)	Allele 2	*ant*(6)-*Ia*, *tet*(O)	0.125
KU62 (dog/pus/ 2021/Tokyo)	BAAAAN000000000.1 (DRR527835)	2,131,326	38	596×	183,463	2,072/2,031/36/2/3	39.6	85.1	ST100 (singleton)	Allele 10	None	0.125
KU66 (dog/ear discharge/ 2021/Kanagawa)	BAAAAL000000000.1 (DRR527836)	2,115,708	69	454×	92,775	2,010/1,968/36/3/3	39.6	84.2	ST46 (CC46)	Allele 2	*ant*(6)-*Ia*, *erm*(B), *tet*(O)	0.06
KU71 (dog/cornea/2021/ Chiba)	BTXR00000000.1 (DRR518367)	2,384,686	76	780×	83,986	2,263/2,233/25/2/3	38.8	84	ST46 (CC46)	Allele 2	*ant*(6)-*Ia*, *erm*(B), *tet*(O)	0.125
KU81 (dog/pus/2021/Tokyo)	BTXQ00000000.1 (DRR518368)	2,054,404	44	950×	93,358	1,956/1,921/30/2/3	39.6	85.1	ST46 (CC46)	Allele 2	*ant*(6)-*Ia*, *erm*(B), *tet*(O)	0.125

^*a*^ Agar plate dilution method was performed as recommended by Clinical and Laboratory Standards Institute document M100-S16.

^*b*^ We used MLST application ver. 2.0 [https://cge.food.dtu.dk/services/MLST/, Center for Genomic Epidemiology (CGE)] with genome sequences.

^*c*^ M-like protein allele typing was conducted based on our previous typing methods (https://doi.org/10.1016/j.vetmic.2018.09.021) (https://doi.org/10.1016/j.jiac.2020.04.004).

^*d*^ ResFinder ver. 4.4.1 (http://genepi.food.dtu.dk/resfinder, CGE) was applied with genome sequences.

^*e*^ We also measured penicillin-G MIC of <0.03 mg/mL against American Type Culture Collection reference strains 12394/12388 *Streptococcus dysgalactiae* subsp. *equisimilis* as the controls. We found AA substitution (S**T**MK to S**A**MK) at a SxxK motif (at AA positions 340–343) within transpeptidase domain in penicillin-binding protein 2× from three KU44/KU62/KU71 strains revealing increased MIC (0.125 mg/mL) as previously described (10). CDS, coding DNA sequence; CRISPR, clustered regularly interspaced short palindromic repeats; NCTC, National Collection of Type Cultures.

Strains were inoculated on sheep blood agar plates and incubated aerobically in 5% CO_2_ at 35°C for 24 h. A β-hemolytic single colony with a gray-white smooth appearance was picked up from plates and grown overnight in Todd–Hewitt broth supplemented with yeast extract. DNAs were extracted using the DNeasy Blood & Tissue Kit (Qiagen, Germany) (6). Whole-genome sequencing was performed on a DNBSEQ-G400RS platform (MGI-Tech, Japan) using DNA nanoball technology (7, 8). The sequencing library by MGIEasy FS DNA Library Prep Set (item no. 1000006987; MGI-Tech) was loaded into FCL flow cell. Paired-end runs were performed with a read length of 2 × 150 bp.

Sequencing yielded total lengths of 3,198,740–7,985,311 reads (0.960–2.396 Gbp). We assessed read lengths/ambiguous bases/quality scores for the quality control. Failed reads with low-quality scores were removed. The reads were trimmed using the quality trimming tool in CLC Genomics Workbench (ver. 8) with default parameters (quality limit/maximum number of ambiguous bases). *De novo* assembly was performed using the CLC Genomics Workbench with modified parameters, wherein the minimum contig length was set to 500 bp. Draft genome sequences were annotated using the DDBJ Fast Annotation and Submission Tool (DFAST; https://dfast.nig.ac.jp) (9). The assembly metrics and annotated features are provided in Table 1.

We found the AA substitution (S**T**MK to S**A**MK) at a SxxK motif (at AA positions 340–343) within the transpeptidase domain in PBP 2× from three KU44/KU62/KU71 strains revealing increased MIC (0.125 µg/mL) as previously described (10). We also determined sequence types using the MLST application ver. 2.0 (https://cge.food.dtu.dk/services/MLST/) (11), M-like protein alleles (12, 13), and acquired antimicrobial resistance genes using ResFinder ver. 4.4.1 (http://genepi.food.dtu.dk/resfinder) (14) (Table 1).

## Data Availability

Seven genome sequences have been deposited in DDBJ/EMBL/GenBank under the nucleotide accession numbers BAAAAO000000000.1, BTXP00000000.1, BAAAAM000000000.1, BAAAAN000000000.1, BAAAAL000000000.1, BTXR00000000.1, and BTXQ00000000.1, with SRA accession numbers DRR527837, DRR518369, DRR527834, DRR527835, DRR527836, DRR518367, and DRR518368.
